# Influence of the SLC6A3-DAT1 Gene on Multifaceted Measures of Self-regulation in Preschool Children

**DOI:** 10.3389/fpsyg.2017.00026

**Published:** 2017-01-19

**Authors:** Lina M. Cómbita, Pascale Voelker, Alicia Abundis-Gutiérrez, Joan P. Pozuelos, M. Rosario Rueda

**Affiliations:** ^1^Department of Experimental Psychology and Mind, Brain and Behavior Research Center (Centro de Investigación Mente, Cerebro y Comportamiento), University of GranadaGranada, Spain; ^2^Department of Psychology, University of OregonEugene, OR, USA

**Keywords:** self-regulation, SLC6A3-DAT1 gene, executive control, preschool age, dopamine

## Abstract

Development of self-regulation, the capacity to voluntarily modulate thoughts, emotions and actions is strongly related to the maturation of the dopamine-mediated executive attention network (EAN). The attention control processes associated with the EAN greatly overlap with efficiency of the executive functions and are correlated with measures of effortful control. Regulation of dopamine levels within the EAN, particularly in the basal ganglia is carried out by the action of dopamine transporters. In humans, the SLC6A3/DAT1 gene carries out the synthesis of the DAT protein. The 10-repeat allele has been associated with an enhanced expression of the gene and has been related to ADHD symptoms. Little is known about the impact of DAT1 variations on children's capacity to self-regulate in contexts that impose particular demands of regulatory control such as the school or home. This study defines a multi-domain phenotype of self-regulation and examines whether variations of the DAT1 gene accounts for individual differences in performance in 4–5 year old children. Results show that presence of the 10r allele is related to a diminished ability to exert voluntary regulation of reactivity. These findings shed light on the neurobiological mechanisms underlying individual differences in self-regulation during childhood.

## Introduction

Self-regulation (SR), the capacity to voluntarily modulate thoughts, emotions, and actions, has been found to positively influence social adjustment as well as cognitive and academic performance from early childhood and adolescence (Checa and Rueda, 2011; Eisenberg et al., 2011) to adulthood (Moffitt et al., 2011).

The development of SR skills is related to the maturation of the executive attention network (EAN), a neural system involving the anterior cingulate cortex (ACC), the dorsolateral prefrontal cortex and the basal ganglia (Posner and Rothbart, 2009). Activation of the EAN occurs when performing attention control tasks as those involving error detection, inhibitory control and conflict processing (Rueda et al., 2005). Also, activation of the ACC is associated with processing of affective information and the regulation of emotional reactivity (Bush et al., 2000). Several studies have reported that performance on tasks that engage the EAN correlates with children's social behavior in the school (Checa et al., 2008) as well as with caregiver scores and self-reported measures of effortful control, a temperamental factor related to SR (see Rueda, 2012 for a review).

The attention control processes associated with the activation of the EAN are known to support higher-level executive functions (EFs) such as cognitive flexibility, planning, problem-solving, and reasoning (Diamond, 2013). Some studies have found that the performance of tasks engaging executive attention processing and higher-level EFs are highly correlated with measures of general intelligence (Brydges et al., 2012; Duggan and Garcia-Barrera, 2015) and exhibit overlapping patterns of activation in the dorsolateral prefrontal cortex (Duncan and Owen, 2000; Duncan et al., 2000).

The development of executive attention skills follows a protracted course that starts in the second half of the first year of life, exhibiting a major growth spurt during the preschool period (Rueda et al., 2005), and continuing during adolescence (Pozuelos et al., 2014). Electrophysiological (Abundis-Gutiérrez et al., 2014) and neuroimaging (Casey et al., 2000; Konrad et al., 2005) studies have shown that structural and functional changes in the brain support this development.

Studying the interplay of cognitive processes supported by the interaction of specific brain networks and neuromodulators provides a method to understand the neurological basis of individual differences in SR. In the forebrain, dopamine (DA) neurotransmission plays a central role in the modulation of the function of basal ganglia structures and its integration with areas in the cerebral cortex. This integrative action is critical for the modulation of processes that underlie the voluntary regulation of behavior including motor reactivity, attention, learning, motivation, reward processing, and higher-order executive functions (Nieoullon, 2002). Thus, DA neurotransmission is considered a key endophenotype for the study of individual differences in the development of the executive processes that conform SR phenotypes.

Regulation of DA levels in the basal ganglia, particularly in the dorsal and ventral striatum, is mainly carried out by the action of dopamine transporters (DAT; Piccini, 2003). In humans, the DAT protein is synthesized from the SLC6A3/DAT1 gene. A 40-bp variable number of tandem repeats (VNTR) in the 3′ untranslated region of the gene has been related to differences in the expression of the gene (Vandenbergh et al., 1992). In particular, the 10-repeat allele has been associated with greater expression of the gene compared to the 9-repeat allele (Fuke et al., 2001), a relation that appears to be positively associated with the number (either none, one, or two) of 10r alleles (Mill et al., 2002). Moreover, density of the DAT binding site is about 50% higher for carriers of the 10r allele, a phenotype that functionally translates into less availability of DA in the synaptic space, and hence hypoactivity of the DA pathways (VanNess et al., 2005; Yang et al., 2007).

At the cognitive and behavioral levels, a large bulk of clinical studies have linked DAT functioning and variations of the SLC6A3/DAT1 gene to ADHD. For instance, several studies have reported an increased density of DAT in children and adults diagnosed with ADHD (Cheon et al., 2003; Krause et al., 2003) and meta-analytic studies have found significant associations between the 10r allele of the DAT1 and ADHD (Yang et al., 2007; Gizer et al., 2009; although see Rommelse et al., 2008 for contradictory results). Neuroimaging studies have also shown that homozygosity for the 10r allele is associated with reduced cortical thickness in the prefrontal cortex in children and adolescents diagnosed with ADHD (Fernández-Jaén et al., 2015).

Despite of all these significant contributions, it is still unclear how variations of the DAT1 gene influence children's SR, particularly in typical development. Most of the studies in the field have approached this question using laboratory assessments of attention control and EFs. Therefore, little is known about the impact that variations of the DAT1 gene may have on children's capacity to implement executive control in contexts that impose particular demands of SR such as the school or home. In this study, we attempt to contribute to this next step by assessing preschoolers' SR taking a multifaceted perspective and examining whether presence of the 10r allele, accounts for individual differences in children's performance. To achieve this goal we have included a variety of tools that allow evaluation of cognitive, temperamental, and socio-emotional aspects of children's SR from different angles.

Three fundamental aspects of the neurobiological basis of SR contributed to formulate our hypothesis: (1) DA circuits connecting the structures of the EAN, in particular the basal ganglia and the prefrontal cortex, have been found to be crucial for the regulation of attention, actions, and emotions (Nieoullon and Coquerel, 2003); (2) regulation of DA levels in the basal ganglia is carried out by the action of DAT (Piccini, 2003); and (3) presence of the 10r allele of the DAT1 gene, presumably following an allele dosage effect (Mill et al., 2002), has been related to a diminished functioning of the dopaminergic transmission system (Fuke et al., 2001; VanNess et al., 2005). Therefore, we hypothesize that the presence of the 10r allele would be related to poorer performance in a battery of attention and EF tasks, as well as lower scores on parents'- and teachers'-reported measures of SR.

## Materials and methods

### Participants

A total of 127 children (55 female) between the ages of 4 and 6 (*M*: 63.9 months; *SD*: 6.5) whose parents gave written consent participated in the study. Parents and teachers were contacted in a wide number of preschools and urban nursery schools in the city of Granada (Spain) and were informed about the general purpose of the study. Only children whose parents agreed to voluntary participation were included in the study. All participants except two (carrying the 10/10 and the 10/9 genotypes) were Caucasian/European. The socioeconomic background of children was measured based on the educational level of the mother. No statistically significant differences were observed between the 10r-Ab and the 10r-Pr groups [*t*_(24.4)_ = −1.47, *p* = 0.15] or between the three different genotype groups [*F*_(2, 113)_ = 2.1, *p* = 0.12]. Participants had normal or corrected-to-normal sensory capacities, no history of chronic illness and/or psychopathologies, and were not under pharmacological treatment of any kind as informed by caregivers. Prior to undertaking the investigation, ethical approval was obtained from the Research Ethics Committee of the University of Granada. Children received a t-shirt with the logo of the lab in appreciation for their participation in the study.

### Procedure

Data of the study were collected over a period of two school years. Children were assessed in two different sessions by the same experimenter with no more than 5 days elapsing between session 1 and 2. The first session took place at the school where children underwent a cognitive assessment that included delay of gratification, and intelligence measures. This session lasted ~30 min including short breaks between tasks. The second session took place within 1 week in the laboratory, where children completed a Flanker and Go/No-Go tasks, and the saliva sample for DNA analysis was collected. While children were carrying out the tasks, their parents were asked to complete a temperament questionnaire and the Emotion Regulation Rating Scale (Carlson and Wang, 2007). Additionally, teachers received a modified version of the teacher-report version of the Health Resources Inventory (HRI; Juvonen and Keogh, 1992), a questionnaire informing about schooling skills, and were instructed to complete it separately for each participant.

### Genotyping procedure

DNA was isolated from saliva samples using Oragene collection kits (DNA Genotek Inc., Ontario, Canada) according to the manufacturer's instructions. Approximately 10–40 ng of template was included in each PCR amplification, reactions contained 0.2 mM each deoxynucleotide, 0.2 μM each oligonucleotide, 0.05 U/μl recombinant Taq DNA polymerase with its 1x reaction buffer (NH_4_)_2_SO_4_ (Thermo Fisher Scientific, Pittsburg, PA), and 8% DAT1QuickExtract buffer V1.0 (Epicentre Biotechnologies, Madison, WI) in addition to PCR-specific optimizations. The DAT1 amplification contained 1.5 mM MgCl_2_, 0.6 M betaine, and the oligonucleotides DAT1F 5′-TGTGGTGTAGGGAACGGCCTGAG and DAT1R 5′-CTTCCTGGAGGTCACGGCTCAAGG (Shinohara et al., 2004). Amplification conditions were the following: 95°C 4 min; 35x 94°C 30 s, 65°C 1 min, 72°C 30 s; 72°C 3 min. Amplified products were size separated on a 2% agarose gel (GenePure LE, BioExpress, Kaysville, UT) and visualized using ethidium bromide. Genotype was grouped by 10/10 (homozygous for the 480 bp 10-repeat product), 10/9 (carriers of one 480 bp product), and 9/9 (homozygous for the 440 bp 9-repeat product). Three participants were excluded from data analysis because they carried rare variants of the DAT1 gene. Numbers and frequencies of alleles and genotypes are presented in Table 1.

**Table 1:** **Genetic distribution of the sample**.

	**DAT1 gene**						
	**Alleles**		**10r Allele**		**Genotype**		
	**10**	**9**	**Pr**	**Ab**	**10–10**	**10–9**	**9–9**
Valid *n*	169	85	110	17	59	51	17
Frequency	0.67	0.33	0.87	0.13	0.47	0.40	0.13

### Materials

#### Teachers' report of schooling skills

A modified version of the Health Resources Inventory (HRI; Juvonen and Keogh, 1992) was used in this study. The number of items included in each scale was reduced to five and the student-role understanding scale was not included for brevity. Instead, we included 5 items about children's assertiveness in order to assess competences in the socio-emotional domain. In total, the questionnaire included 20 items taping teachers' perceptions of children's school competence in four dimensions: Assertiveness (Cronbach's α = 0.84), sociability (α = 0.82), rule following (α = 0.90, after the removal of one item that caused poor internal consistency), and frustration tolerance (α = 0.70). We calculated the mean score for each scale as well as the averaged mean of all of them (Schooling Skills, α = 0.87).

#### Parents' report of temperament and emotional regulation skills

##### Children's behavior questionnaire (CBQ–Putnam and Rothbart, 2006)

The CBQ was utilized to collect information on children's effortful control reported by parents. Participants enrolled in the first year of the study (*n* = 36) completed the long version of the CBQ (195 items), while the rest of participants (*n* = 95) completed the short version of the CBQ (94 items). In order to equal the number of items included in the scales, we included only the items completed by all participants (i.e., some items of the long CBQ version were excluded from the analyses). The effortful control (EC) factor of the CBQ provides a measure of children's capacity to voluntarily regulate emotions and actions through five scales: Inhibitory control, attentional focusing, low-intensity pleasure, perceptual sensitivity, and smiling and laughter. In order to increase the internal consistency of the different scales, one item was removed from the low-intensity pleasure, perceptual sensitivity, and smiling and laughter scales. After this process, reliability was modest for the inhibitory control (α = 0.62), perceptual sensitivity (α = 0.68), and smiling and laughter (α = 0.61) sub-scales, and appropriate for attentional focusing (α = 0.70), low-intensity pleasure (α = 0.70), and the EC factor (α = 0.79).

##### Emotion regulation rating scale (Carlson and Wang, 2007)

This is a brief questionnaire in which caregivers are asked to answer 6 questions about their children's capacity to modulate their behavior in situations that elicit emotional reactivity. In this study, the internal consistency of the scale was low (α = 0.46) and therefore this measure was not included in the gene variation analyses.

#### Battery of laboratory tasks

##### Delay of gratification (DoG)

We administered a modified version of the DoG task designed by Thompson et al. (1997). Children were instructed to choose between getting one prize immediately or waiting until the end of the task in order to (a) get two prizes (“delay for oneself” condition) or (b) get one prize for themselves and a second one to be given to the experimenter (“social delay” condition). When children decided not to delay they got the prize immediately and were free to use it, whereas in trials in which they chose to delay the prize was introduced in an envelope that was only given to the child at completion of the task. The envelope was kept on one side of the table during the realization of the task. The delay period was ~10 min, which corresponded to the time children took to complete the DoG task on average. Options were presented to participants using a structured sentence in which the delay option was presented first (i.e., “do you want to get two prizes at the end of the game or one prize now?” vs. “do you want to get one prize now or two prizes at the end of the game?) in 50% of the trials. Trials of the “delay for oneself” condition were presented first and this order was the same for all participants. We registered the overall percentage of delay choices, as well as separately for each condition.

##### Go/No-Go task

We used a computer-based task in which a traffic light was presented in the center of the screen. Children were instructed to press a key whenever the green light was on (“Go” trials), while inhibiting the response when the red light was on (“No-Go” trials). Children completed 160 trials (25% NoGo), divided into two blocks. Performance for each participant was measured using the Signal Detection Theory (SDT) sensitivity index (*d')* which takes into account both hits and false alarms rates (*d'* = Z score False Alarms–Z score Hits).

##### Flanker task

We used a child-friendly flanker task with square and round shapes (described in Checa et al., 2014). A flanker interference conflict score was calculated using reaction times (Conflict-RT) and percentage of errors (Conflict-Err) by subtracting reaction times and percentage of errors in the congruent condition from that of the incongruent condition. An index of flanker interference was calculated by averaging the *Z* scores for the two measures.

### Intelligence test

#### Kaufman brief intelligence test (K-BIT; Kaufman and Kaufman, 1990)

This test provides standardized scores for two subscales: Verbal and Fluid Intelligence (IQ-v and IQ-f, respectively).

## Results

Pearson correlations analyses were conducted among the variables included in the study. Correlation coefficients are presented in Table 2. Teacher-reported schooling skills correlated positively with parent-reported measures of EC and children's overall percentage of delay choices in the DoG task. Also, a negative correlation was observed between schooling skills and the conflict index obtained with the flanker task, which indicates that higher scores on schooling skills are associated with greater executive attention efficiency (i.e., lower interference costs). Moreover, parent-reported measures of children's EC showed a positive correlation with parents' reports on the emotion regulation scale as well as with children performance on the Go/No-Go task, and the fluid intelligence score. Finally, the flanker conflict index correlated negatively with both verbal and fluid intelligence indexes (crystallized and fluid), that is, lower conflict-interference costs were associated with higher IQ scores.

**Table 2:** **Statistically significant results from Pearson correlation analysis among all dependent variables included in the study**.

				**1**	**2**	**3**	**4**	**5**	**6**	**7**	**8**	**9**	**10**	**11**	**12**	**13**	**14**	**15**	**16**
Teachers' Reported	Schooling Skills	1.	Schooling skills composite	–															
		2.	Assertiveness	0.54^***^	–														
		3.	Sociability	0.82^***^	0.29^**^	–													
		4.	Rule Following	0.84^**^	–	0.68^***^	–												
		5.	Frustration Tolerance	0.68^***^	–	0.43^***^	0.59^***^	–											
Parents' Reported	Temperament	6.	Effortful control	0.29^*^	0.25^*^	0.26^*^	0.25^*^	–	–										
		7.	IC	0.38^***^	–	0.27^*^	0.39^***^	–	0.65^***^	–									
		8.	AF	0.28^*^	0.22^*^	0.24^*^	0.27^*^	–	0.74^**^	0.48^***^	–								
		9.	LIP	–	–	–	–	–	0.56^***^	–	0.33^***^	–							
		10.	PS	–	–	–	–	–	0.61^***^	0.22^*^	0.22^*^	0.22^*^	–						
		11.	S&L	–	–	–	–	–	0.65^***^	0.18^*^	0.26^**^	0.34^***^	0.30^**^	–					
Battery of Lab Tasks	DoG	12.	% Delay choices	0.23^*^	–	0.27^*^	–	0.27^*^	–	–	–	–	–	–	–				
	Go-NoGo	13.	*d*'	–	–	–	–	–	0.26^*^	0.21^*^	0.22^*^	–	–	0.23^*^	–	–			
	Flanker	14.	Conflict z-index	−0.24^*^	−0.25^*^	–	−0.24^*^	–	–	–	–	–	–	–	–	–	–		
Intelligence	IQ	15.	Verbal	–	–	–	–	–	–	–	–	–	–	–	–	–	−0.24^**^	–	
		16.	Fluid	–	0.24^*^	–	–	–	0.26^**^	–	0.28^**^	–	–	0.19^*^	–	–	−0.22^*^	0.35^***^	–

Asterisks denote the level of significance for each correlation

*** p < 0.001;

** p < 0.01;

* *p < 0.05*.

### DAT1 modulation

A series of one-way analyses of variance (ANOVA) and linear contrasts analyses were conducted to test whether indexes of tasks performance, reported schooling skills and EC scores vary according to the number of 10r alleles (i.e., 2, 1, or 0) carried by the children. Therefore, for each ANOVA, children's scores of the different measures were included as the dependent variable while the three allelic combinations of the DAT1 gene (10/10, 10/9, 9/9) were included as the Genotype Group factor. Additionally, we conducted a series of independent-samples *t*-test to compare all scores included in the study in function of the presence vs. absence of the 10r allele (10r-Pr vs. 10r-Ab). Preliminary analyses showed statistically significant differences between genotype groups in variances of most of the dependent measures. This was somewhat expected given the large n differences between groups in our sample related to known differences in the frequency of the different variations of the DAT1 gene found in Caucasian and African American populations (Vandenbergh et al., 1992). Therefore, as a matter of caution we assumed unequal variances on all subsequent analysis. Also, we calculated the effect size (ES) of the between-group differences for all dependent variables using the pooled standard deviation of the groups (Mean_Pr_ − Mean_Ab_/Pooled SD_Pr&Ab_). A summary of the results including size of the sample on each task, means, *SD*, and *p*-values for all dependent variables is presented in Table 3. Differences in valid sample sizes for different dependent variables are mainly due to the inclusion of new measures in the second year of the study (emotion regulation rating scale, Go/No-Go task and the HRI). Also, some parents and teachers did not complete all the questionnaires.

**Table 3:** **Summary of results on each task/questionnaire and genotypes used in our study**.

**Questionnaire/Task**		**DV**	**Mean (*****SD*****)**			**One-way ANOVA**	**Linear contrast**	**Mean (*****SD*****)**		***t*-test**
			**10/10**	**10/9**	**9/9**			**10r-Pr**	**10r-Ab**	**Pr vs. Ab**
Teachers' reported	Schooling skills	*N*	38	28	14			66	14	
		Schooling skills composite (0.87)	3.5 (0.42)	3.7 (0.52)	3.9 (0.51)	4.04^*^	7.37^**^	3.6 (0.47)	3.9 (0.51)	−2.12^*^
		Assertiveness (0.84)	3.7 (0.74)	3.8 (0.67)	3.9 (0.86)	<1	1.01	3.7 (0.71)	3.9 (0.87)	−0.65
		Sociability (0.82)	3.6 (0.53)	3.8 (0.70)	4.1 (0.56)	3.45^*^	6.75^*^	3.7 (0.61)	4.1 (0.56)	−2.42^*^
		Rule following (0.90)	3.7 (0.71)	3.8 (0.84)	4.2 (0.75)	1.79	*3.59*^#^	3.8 (0.76)	4.2 (0.75)	−*1.84*^#^
		Frustration tolerance (0.70)	3.2 (0.56)	3.4 (0.57)	3.5 (0.58)	3.21^*^	4.74^*^	3.2 (0.71)	3.4 (0.65)	−1.13
Parents' reported	Temperament	*N*	55	48	17			103	17	
		Effortful control (0.79)	5.2 (0.51)	5.2 (0.65)	5.3 (0.41)	<1	<1	5.3 (0.59)	5.4 (0.43)	−1.30
		Inhibitory control (0.62)	4.6 (0.87)	4.9 (0.93)	4.8 (0.97)	1.24	<1	4.8 (0.90)	4.8 (0.97)	−0.03
		Attentional focusing (0.70)	4.5 (1.01)	4.6 (1.15)	5 (0.80)	1.65	*3.30*^#^	4.6 (1.07)	5.0 (0.80)	−2.13^*^
		Low intensity pleasure (0.70)	6.1 (0.60)	6.1 (0.72)	6.0 (0.56)	<1	<1	6.1 (0.66)	6.0 (0.56)	−0.22
		Perceptual sensitivity (0.68)	5.8 (0.87)	5.7 (1.0)	5.9 (0.79)	<1	<1	5.7 (0.93)	5.9 (0.79)	−0.59
		Smiling and laugher (0.61)	5.4 (0.94)	5.2 (1.02)	5.5 (0.72)	<1	<1	5.3 (0.98)	5.5 (0.72)	−1.04
Battery of lab tasks	DoG	*N*	59	51	17			110	17	
		% Delay choices	56.1 (30.7)	58.6 (32.0)	69.2 (18.7)	1.26	2.53	57.2 (31.2)	69.2 (18.7)	−2.20^*^
	Go/No-Go	*N*	44	26	15			70	15	
		*d*'	1.2 (0.67)	0.9 (0.53)	1.2 (0.68)	1.22	<1	1.1 (0.63)	1.2 (0.68)	−0.51
	Flanker	*N*	54	48	15			102	15	
		Conflict z-index	0.09 (0.7)	0.08 (0.7)	−0.25 (0.7)	1.46	2.68	0.09 (0.7)	−0.25 (0.7)	*1.78*^#^
Intelligence	IQ	*N*	58	51	17			109	17	
		Verbal	107 (16.3)	108 (12.8)	105 (11.9)	<1	<1	107 (14.7)	105 (11.9)	0.70
		Fluid	105 (12.2)	104 (11.8)	110 (9.6)	1.60	2.20	104 (12.0)	110 (9.6)	−2.03^*^

Battery of lab tasks: DoG, Delay of Gratification Task; Conflict z-index: Mean Z scores = Reaction Time-_ConflictEffect_ + % Errors-_ConflictEffect_ (Conflict effect: Incongruent − Congruent). Cronbach's Alpha is shown in brackets for each scale. Asterisks denote the level of significance

** p < 0.01;

* p < 0.05;

# *p < 0.1. Bold values depict statistically significant comparisons. Italic values depict marginally significant comparisons*.

#### Teachers' reported schooling skills

##### Schooling skills

**Figure 1:**
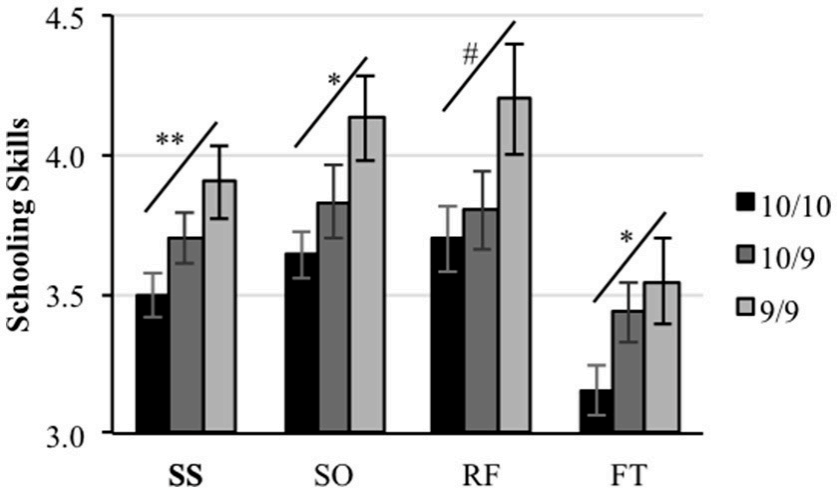
**Allele-dosage effect**. Teachers' reported schooling Skills. Schooling skills composite (SS), socialization scale (SO), rule following scale (RF), and frustration tolerance scale (FT). Asterisks represent significance of the linear contrast. **p < 0.01; *p < 0.05; ^#^*p* < 0.1.

One-way ANOVAs and linear contrasts revealed a statistically significant allele-dosage effect for the schooling skills composite [*F*_(2, 77)_ = 4.04, *p* = 0.02; *linear contrast*: *F*_(1, 77)_ = 7.37, *p* = 0.008]; the sociability scale [*F*_(2, 77)_ = 3.45, *p* = 0.04; *linear contrast: F*_(1, 77)_ = 6.75, *p* = 0.01] and the frustration tolerance scale [*F*_(2, 77)_ = 3.21, *p* = 0.04; *linear contrast: F*_(1, 77)_ = 4.75, *p* = 0.03], where children carrying two copies of the 10r allele (10/10) scored lower than those carrying only one copy (10/9) who in turn obtained lower scores than children who carried zero copies (9/9) of the allele (see Figure 1). A similar but non-significant trend was observed for the rule following scale [*F*_(2, 77)_ = 1.79, *p* = 0.17], however, the linear contrast analysis did show a marginally significant linearity for this trend *F*_(1, 77)_ = 3.59, *p* = 0.06. No significant differences between the three genotypes were found for the assertiveness scale (*F* < 1).

**Figure 2:**
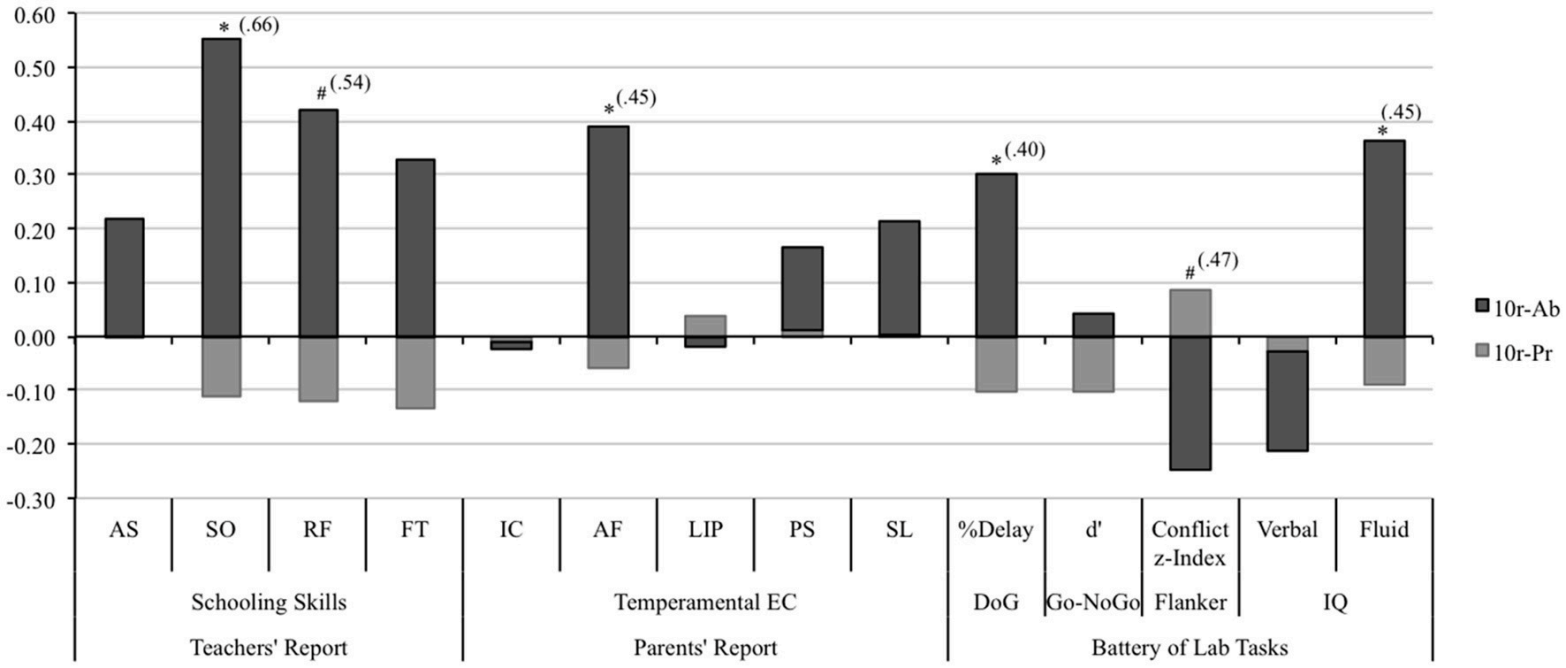
**Performance comparison for the 10r-Pr and the 10r-Ab groups using the Z-scores of the dependent variables**. *Teachers' reported schooling skills:* Assertiveness (AS), socialization (SO), rule following (RF), and frustration tolerance (FT). *Parents' reported temperament:* inhibitory control (IC), attentional focusing (AF), low intensity pleasure (LIP), perceptual sensitivity (PS), and smiling and laughter (S&L). *Battery of laboratory tasks:* Delay of Gratification Task (DofG), Go-NoGo task: *d'* = Z score False Alarms–Z score Hits; Flanker Task: Conflict z-index = (Z scores) Conflict-RT + (Z scores) Conflict-Errors (Conflict effect: Incongruent − Congruent). Asterisks represent significance of the independent-samples *t*-test: *p < 0.05; ^#^*p* < 0.1 The size effect of the 10r-Pr vs. 10r-Ab comparison for those measures where the *t*-test was statistically significant is presented in brackets. Size Effect = MeanPr − MeanAb/Pooled SD Pr&Ab.

An independent-samples *t*-test revealed that children carrying one or two copies of the 10r allele (10r-Pr) were given statistically significant lower ratings on the schooling skills composite [*t*_(17.9)_ = −2.12, *p* = 0.05; ES = 0.61] and the sociability scale [*t*_(20)_ = −2.42, *p* = 0.03; ES = 0.66], and marginally significant ratings on the rule following scale [*t*_(19.1)_ = −1.84, *p* = 0.08; ES = 0.54] compared with those homozygous for the 9r allele (see Figure 2). No statistically significant differences were found for the frustration tolerance [*t*_(18.9)_ = −1.57, *p* = 0.13; ES = 0.46] or the assertiveness scales [*t*_(16.9)_ = −0.65, *p* = 0.52; ES = 0.22]. When the traditional 10/10 vs. 10/9 + 9/9 comparison is tested, statistically significant differences are also found for the schooling skills composite [*t*_(77.1)_ = 2.54, *p* = 0.01], the sociability scale [*t*_(78)_ = 2.12, *p* = 0.04], and the frustration tolerance scale [*t*_(78)_ = 2.49, *p* = 0.01]. Conversely, the same comparison, which assumes dominance of the 9r allele, did not show statistically significant differences for the rest of the dependent variables (all comparisons *p* > 0.05).

#### Parent's reported EC

##### Children's behavior questionnaire

A tendency toward an allele-dosage effect was observed for the attentional focusing scale, however, the one-way ANOVA did not reach significance levels, [*F*_(2, 117)_ = 1.65 *p* = 0.19; *linear contrast: F*_(1, 117)_ = 3.30, *p* = 0.07]. Differences between the three genotypes showed the same tendency described above: An increasing number of 10r alleles was associated with lower scores on the attentional focusing scale. Comparisons between the 10r-Pr vs. 10r-Ab groups revealed that carriers of the 10r allele (either one or two copies) obtained significantly lower scores in the attentional focusing scale compared to children who did not carry the allele [*t*_(26.6)_ = −2.13, *p* = 0.03; ES = 0.45] (see Figure 2). No statistically significant differences emerged for the remaining scales (for all comparisons *p* > 0.05).

#### Performance of laboratory tasks

##### Delay of gratification task

No statistically significant differences were observed when the delay responses were analyzed independently for each condition (for all comparisons *p* > 0.05). Regarding the overall percentage of delay of gratification responses, ANOVAs and linear contrast did not reveal statistically significant differences between the genotypic variations [*F*_(2, 124)_ = 1.26, *p* = 0.29; *linear contrast: F*_(1, 124)_ = 2.52, *p* = 0.11]. However, comparisons between the 10r-Pr and the 10r-Ab groups revealed that children carrying one or two copies of the 10r allele showed a lower percent of overall delay of gratification responses compared to those who did not carry the allele [*t*_(31.8)_ = −2.20, *p* = 0.04; ES = 0.40].

##### Flanker task

Genotype comparisons did not support presence of an allele dosage effect for the flanker task [*F*_(2, 114)_ = 1.46, *p* = 0.24; *linear contrast: F*_(1, 114)_ = 2.68, *p* = 0.10]. However, a marginally significant difference was found for the conflict-processing index when the 10r-Pr and the 10r-Ab groups were compared. Children in the 10r-Pr group exhibited larger interference costs compared with those in the 10r-Ab group [*t*_(18.9)_ = 1.78, *p* = 0.09; ES = 0.47].

##### Go/No-Go task

No statistically significant genetic-based differences were observed on children's performance of the task when the three genotypes were compared [*F*_(2, 82)_ = 1.23, *p* = 0.30; *linear contrast: F* < 1]. Similarly, no statistically significant differences emerged between carriers and non-carriers of the 10r allele [*t*_(19.5)_ = −0.51, *p* = 0.62; ES = 0.15).

##### Intelligence

One-way ANOVAs and linear contrasts revealed no statistically significant effects for the fluid-IQ [*F*_(2, 123)_ = 1.60, *p* = 0.20 linear contrast: *F*_(1, 123)_ = 2.20, *p* = 0.14] or the verbal-IQ (*F* < 1; linear contrast: *F* < 1). Further analysis addressing differences in the performance of 10r carriers and non-carriers revealed that 10r-Ab group outperformed those who carried one or two copies of the 10r allele [*t*_(24.4)_ = −2.03, *p* = 0.05; ES = 0.45) on the fluid IQ test (see Figure 2). No significant differences were found between the 10r-Pr and the 10r-Ab groups on the verbal IQ scale (*p* = 0.49).

## Discussion

The main goal of the present study was to determine whether DAT1 contributes to individual differences in children's SR skills. We evaluated children's SR taking a multifaceted perspective, which included parents' and teachers' reports, as well as a battery of cognitive tasks involving delay of gratification, conflict processing, inhibitory control, and intelligence assessments.

In line with our hypotheses, correlation analyses revealed that school, home, and lab observations of SR were interrelated. We found that independent reports of schooling skills and EC, respectively reported by teachers and parents, were positively related to one another. This finding supports previous evidence reported by Checa et al. (2008) with 12-year-old children and suggest that as early as 4 years of age, temperamental EC is central to schooling adjustment in both social and academic domains, as children with higher EC were rated better in socialization and learning-promoting behaviors in the school context.

On the question of whether adult-reported measures of SR relate to children's performance on the battery of lab tasks, we found that teacher-reported schooling skills, particularly the sociability and the frustration tolerance scales were positively related with the capacity of children to delay gratification. This correlation indicates that the ability to regulate reactivity in situations that involve highly rewarding motivational states is related to the adjustment of children in the school. This result has been largely documented in the literature; in fact, the ability to delay gratification in the preschool period is a powerful predictor of outcomes in the social, cognitive, and health domains later in life (Mischel et al., 2011; Moffitt et al., 2011). Furthermore, we found that children with lower flanker interference scores (i.e., better executive attention efficiency) also exhibited higher scores on the verbal and fluid intelligence test, a result that reflects the contribution of executive attention mechanisms to the performance of intelligence tests. Likewise, children who exhibited better performance on the flanker task were perceived as better adjusted in school by their teachers. This result is consistent with those reported by Checa et al. (2008) and confirms that executive attention skills play a central role in social and academic school adjustment in children as young as 5 years of age.

Furthermore, we found that parent-reported attentional focusing is related to children's inhibitory control in the Go-NoGo task and fluid IQ score. This finding is consistent with previous research documenting the connection between efficiency of the EAN and aspects of temperamental EC during childhood (see Rueda, 2012). Also, previous studies have reported an association between reasoning skills and preschoolers' EC (Kochanska et al., 2007). The construct of fluid intelligence comprises high-level EFs such as problem solving and reasoning skills, which rely to a large extent on the efficiency of executive control processes including inhibitory control and cognitive flexibility (Diamond, 2013); a connection that is supported by the largely overlapping anatomy between the EAN and regions activated when performing general intelligence tasks (Duncan et al., 2000).

### Role of the DAT1 gene on individual differences in SR

Results reported in the current study provide a new insight on the neurobiological mechanisms underlying SR in childhood. While previous studies aimed to characterize the number of biochemical pathways that need to be suboptimal before a particular phenotype emerge (i.e., ADHD), in the present study we aimed at understanding whether inefficiency of a dopamine pathway influences a well-defined phenotype, particularly when typical development takes place.

Based on the findings reported by Mill et al. (2002), we first explored whether the influence of the 10r allele exhibits an allele dosage effect. Specifically, we proposed that there is a dose-related effect associated with the level of synaptic DA due to overall DAT activity, where greater synaptic DA is associated with better cumulative performance. This would be evidence of haploinsufficiency, where there is incomplete dominance of the 10r allele. Interestingly, since we consider this effect of DAT activity to be striatal, our results indicate that optimal performance is achieved through relatively high synaptic DA levels, unlike that seen in prefrontal-mediated performance that follows an inverse *U*-shaped curve for optimal DA levels (Williams and Goldman-Rakic, 1995). This quantitative effect of the 10r allele was statistically significant for the teacher-reported measures of socialization and frustration tolerance, and marginally significant for rule following. Results showed that carriers of two copies of the 9r allele performed better than those who carry only one copy of the allele, who in turn over performed those who are homozygous for the 10r allele. The same tendency, albeit not statistically significant, was observed in the attentional focusing scale as well as in the delay of gratification tasks, the flanker task and the fluid IQ sub-scale.

Due to the low incidence of individuals homozygous for the 9r allele, most of the previous studies in the field have compared the performance of individuals with the 10/10 homozygous genotype with that of individuals with the 10/9 heterozygous genotype, which assumes dominance of the less frequent 9r allele, for example, as a protective factor for the development of pathologies involving self-regulation deficits such as ADHD. However, as mentioned in the introduction, no conclusive results in favor of such a model have been reported, and studies with healthy individuals have led to contradictory results (see Rommelse et al., 2008 for a review). Nevertheless, our analysis tested this approach; we obtained significant differences in the ANOVA testing the three genotype groups (i.e., 10/10, 10/9, and 9/9) only for schooling skills, and this effect was driven by higher sociability and tolerance to frustration in 9r carriers compared to 10/10 homozygous, a result that may reflect the allele-dosage effect observed for those measures. However, no statistically significant differences were found in the rest of the dependent variables. In contrast to the 9r present vs. absent approach, the current study tests how presence of the 10r allele, a variation that is associated with less efficient DA pathways (Mill et al., 2002; VanNess et al., 2005), influences children's SR. In our analyses, we allowed the possibility that the less frequent 9r allele is recessive and two copies of it are necessary to facilitate more effective DA activity through slower re-uptake in the basal ganglia, which in turn impacts the whole dopaminergic system through the different DA pathways. Our data suggest that children carrying at least one copy of the 10r allele exhibit poorer self-regulatory skills in an array of measures that assess the construct from different angles: Teacher's reported schooling skills, parents' reported attentional focusing and children's performance in the delay of gratification task, as well as a trend in the flanker task. Furthermore, children in the 10r-Pr group exhibited lower scores in fluid IQ, which indicates poorer higher-level executive functions such as abstract reasoning and problem solving.

The fact that statistically significant differences were observed in some of the sub-scales and not in others is also informative to the goals of the present study. For the teacher-reported scale of schooling skills, data revealed differences between genotypes in all sub-scales except the assertiveness one. Likewise, results on the parent-reported measure of Effortful Control reveal genotype-related differences in the Attentional Focusing sub-scale but not in others. From a theoretical perspective, this may be related to some of the dimensions of the Schooling Skills and Effortful Control constructs relying on self-regulation more than others. For instance, following school rules or focusing attention may be more dependent on self-regulatory skills than being assertive or experiencing pleasure out of low-intensity activities. Additionally, results also show consistency across measurements. Thus, in consonance with the results on the parent-reported assessment, the lab measure of inhibitory control (Go-No-go task) does not yield significant performance differences between genotype groups, despite inhibitory control being considered an important component of self-regulation.

These results, together with the correlations found among the measures included in this study, suggest that presence of the 10r allele is related to preschoolers' ability to modulate reactivity in two different but closely related domains. On the one hand, the attention focusing scale and the flanker task assess children's tendency to maintain attentional focus upon task-related channels, a type of regulation that involves the implementation of the so-called cool or neutral cognitive control mechanisms. On the other hand, the schooling skills scale and the delay of gratification task assess children's ability to regulate responses in situations where both reactivity and the modulation of it involve affective and motivational states that are highly influenced by social and appetitive rewards.

At the level of neural functioning, a large number of studies support the idea of separate frontal systems underlying SR in those neutral vs. motivational/emotional-relevant contexts (Zelazo and Cunningham, 2007). While the cool or purely cognitive processes appear to be mostly supported by the activation of dorsolateral and medial structures of the prefrontal cortex, regulation of appetitive/motivated reactivity relies on the activation of a ventromedial network that comprises orbitofrontal structures of the prefrontal cortex (Zelazo and Cunningham, 2007; Perlman and Pelphrey, 2011). Other researchers have also argued for a similar cognitive/neutral vs. emotional division within the ACC (Bush et al., 2000). Although considered as separate systems, activation of those control-related areas greatly depends on the dopaminergic signaling coming from the basal ganglia, particularly the striatum where the highest density of dopamine transporters is located (Piccini, 2003). Some authors have argued that the prime anatomical position of the striatum in the brain circuitry makes of this structure a central node in the loop that connects the cortex with structures in the midbrain where DA-containing cells originate (Graybiel, 2000; Mega et al., 1997). Also, previous studies have shown that striatum DA signaling provides a “gating” mechanism that modulates the access of inputs to the prefrontal systems that support cognitive control (Braver and Cohen, 2000; Potts et al., 2006).

The influence of the 10r allele on children's self-regulatory skills observed in this study may be explained by the patterns of connectivity between both the dorsolateral and ventromedial networks and the striatum. According to the model of DA system regulation and its relation to goal-directed behavior proposed by Grace et al. (2007), DATs play a crucial role specifically on the regulation of phasic DA release which is known to transmit functionally relevant signals related to rewards and the modulation of intentional behavior. The evidence presented in this study indicates that variations of the DAT1 gene relate to individual differences in children's capacity to modulate their behavior. However, further neuroimaging studies need to be undertaken in order to understand how the decreased striatal DA levels linked to the presence of the 10r allele influences the actual functioning of the corticostriatal circuit.

## Conclusions

The current study provides additional evidence regarding the influence of the DAT1 gene on SR during the preschool period. In particular, we found that presence of the 10r allele is associated with preschooler's self-regulatory skills. However, given the small sample size and the moderate internal consistency of some of the measures conclusions must be drawn with caution. Investigating the influence of the DAT1 gene on SR during childhood is an important avenue for further research, which would benefit from including extended sample sizes that would allow a fair balance of genotypes.

In line with Greene et al. (2008) who suggest that the study of the genetic basis of cognitive processes require the precise definition of phenotypes, the assessment of SR carried out in our study was multifaceted and included a wide range of measures provided by different informants and domains. The results presented here suggest that differences in the homeostatic tone of the dopaminergic system in the brain as a consequence of the concentration of dopamine transporters within the striatum, may act as the key endophenotype that allows tracing the link between genetic variability in the SLC6A3-DAT1 gene and individual differences in children's self-regulatory capacities. Therefore, future work will also benefit from combining multifaceted measures of SR with neuroimaging technology in order to confirm the association reported here and identify the neural endophenotype that links DAT1 variations with the observable self-regulatory abilities.

## Ethics statement

Ethical approval was obtained from the Research Ethics Committee of the University of Granada prior to undertaking the investigation. Participation of minors was subject to informed written consent provided by parents/legal tutors.

## Author contributions

MR and LC designed the study. LC, AA-G, and JP were involved in the acquisition and processing of data. PV conducted the genotyping and contributed to the interpretation and writing of the paper. LC and MR were responsible for drafting the work and revising it for intellectual content. All authors provided final approval of the version to be published.

### Conflict of interest statement

The authors declare that the research was conducted in the absence of any commercial or financial relationships that could be construed as a potential conflict of interest. The reviewer EP declared a shared affiliation, though no other collaboration, with the author PV to the handling Editor, who ensured that the process nevertheless met the standards of a fair and objective review.
